# Chitosan/Poly(vinylpyrrolidone) Matrices Obtained by Gamma-Irradiation for Skin Scaffolds: Characterization and Preliminary Cell Response Studies

**DOI:** 10.3390/ma11122535

**Published:** 2018-12-13

**Authors:** Maria Helena Casimiro, Susana R. Gomes, Gabriela Rodrigues, João Paulo Leal, Luís M. Ferreira

**Affiliations:** 1 Centro de Ciências e Tecnologias Nucleares (C2TN), Instituto Superior Técnico, Universidade de Lisboa, Estrada Nacional 10, 2695-066 Bobadela, LRS, Portugal; srrg@fct.unl.pt (S.R.G.); jpleal@ctn.tecnico.ulisboa.pt (J.P.L.); ferreira@ctn.tecnico.ulisboa.pt (L.M.F.); 2 Centro de Ecologia, Evolução e Alterações Ambientais (cE3c) and Departamento de Biologia Animal, Faculdade de Ciências, Universidade de Lisboa, Campo Grande, 1749-016 Lisboa, Portugal; mgrodrigues@fc.ul.pt; 3 Centro de Química Estrutural (CQE), Instituto Superior Técnico, Estrada Nacional 10, 2695-066 Bobadela, LRS, Portugal

**Keywords:** chitosan, poly(vinylpyrrolidone) (PVP), porous scaffolds, γ-radiation, skin regeneration

## Abstract

Several studies have shown that chitosan possesses characteristics favorable for promoting dermal regeneration and accelerated wound healing. In this work we have reported the work that has been done on the development and characterization of biocompatible and biodegradable chitosan based matrices to be used as skin scaffolds. Poly(vinylpyrrrolidone) (PVP) was used as copolymer and a two steps methodology of freeze-drying and gamma irradiation was used to obtain the porous matrices. The influence of PVP content, synthesis procedure and absorbed radiation dose on matrices’ physical, chemical and structural properties was evaluated by ATR-FTIR, TGA, SEM, contact angle measurements and degradation behavior. The in vitro cellular viability and proliferation of HFFF2 fibroblast cell line was analyzed as a measure of matrices’ biocompatibility and ability to assist skin regeneration. Results show that over the studied range values, gamma-radiation dose, copolymer concentration and synthesis procedure can be used to tailor the matrices’ morphology in terms of porosity and surface roughness. Early results from biological assays evidence the biocompatibility of the prepared chitosan/PVP matrices since cells adhered to the surface of all matrices (chitosan/PVP (5%) γ-irradiated at 10 kGy presents the higher cellular viability). These features show that the resultant matrices could be a potential suitable scaffold for skin tissue regeneration.

## 1. Introduction

**Figure 1:**
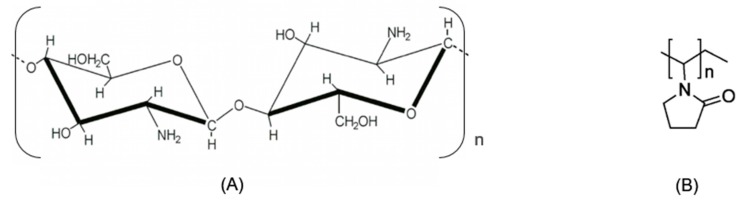
Chemical structure of: (**A**) chitosan; (**B**) Poly(vinylpyrrolidone) (PVP).

Natural polymers are quite common and include polysaccharides, like starch, cellulose and chitin and their derivatives. They exist in a great variety of environments including in our bodies (e.g., DNA and RNA can be considered natural polymers since they contain backbones based on sugar units). The fact that a natural polymer has almost the same structure as the molecules in biological organs results in reducing the chance of rejection by the immune system [1]. Beyond that, skin scaffolds should also be bioresorbed by the body, capable of mimicking structure and biological functions of the extracellular matrix, as well as to provide a good environment for the cells to easily attach, proliferate and differentiate [2,3,4]. Chitosan (Figure 1A), a linear aminopolysaccharide composed of glucosamine and N-acetyl glucosamine units linked by β (1-4) glycosidic bonds formed by N-deacetylation of chitin, has been extensively used in the biomedical area as it exhibits diverse properties that open up a wide-range of applications in biomedical sciences [5,6,7,8].

Poly(vinylpyrrolidone), PVP, (Figure 1B) presents typically as a white to light yellow amorphous powder, soluble in water and in other polar solvents. It has a good human body tolerance and is used in many medical, biomedical and pharmaceutical applications (e.g., plasma volume expander, binder in pharmaceutical tablets, external surgical disinfectants, adhesives, dialysis membranes, contact lenses solutions, toothpastes, etc) extensively reported in literature [9,10]. It is a good emulsifier/stabilizer agent and due to its excellent wetting properties, PVP readily forms homogeneous and resistant films.

Despite the fact that other studies with chitosan/poly(vinylpyrrolidone) using various ways had been reported [11,12] the gamma radiation has several advantages over more conventional technologies: it is a simple, clean and repeatable process; almost any polymer can be used and the degree of advance of the reaction can be easily controlled [13]. In comparison with chemical or photo-induced methods, radiation does not require heating of the system (allowing the use of very sensitive monomers) and does not introduce other species in the process as initiators or photo-sensitizers most of them potentially harmful for human health [14,15]. Thus, materials obtained by γ-radiation processing techniques may present better results in terms of cell viability and proliferation features than the ones prepared by more traditional techniques. Moreover, it offers the possibility of preparation and sterilization in just one single step. The experience of some of the authors follows the same reasoning [16].

Radiation processing techniques has as physical basis the interaction of radiation with matter which can promote specific chemical reactions. As an example, gamma irradiation, that can be considered a clean and environmentally friendly technology (since there is no need of solvents, initiators or high temperatures, avoiding all kind of residues) has been applied over the years with high success in the preparation and modification of polymers. By tuning the experimental conditions like irradiation method, dose rate, irradiation atmosphere, samples’ absorbed dose, reactants’ concentration, etc, it is possible to control the functionalization of polymeric based materials, tailoring its properties and adapting them to different uses like biodiesel production and biomedical applications (mainly through polymerization, crosslinking and/or grafting reactions) [17,18,19,20].

In this work, the methodology that our team has been carrying out on the development/functionalization of biocompatible and biodegradable chitosan/PVP matrices to be used as skin scaffolds, using gamma-radiation as a modifying tool, is further developed. These scaffolds are expected to mimic the extracellular matrix and act as a necessary template to host cell infiltration and to provide the physical and biochemical support in order to guide cells into the tissue. A preliminary cell-scaffold interaction study is also presented.

## 2. Materials and Methods

### 2.1. Materials

Medium molecular weight chitosan, Chit, (190-375 kDa, 75%–85% deacetylated chitin) from Sigma-Aldrich (Darmstadt, Germany) and N-vinyl 2-pyrrolidone (N-VP), (stabilized 98%) from Acros Organics (Geel, Belgium) were used as raw materials and used as received. Acetic acid (min 99.8%, PA) from Riedel-de Haën (Munich, German), was used as chitosan solvent.

### 2.2. Preparation of Chitosan/Poly(vinylpyrrolidone) Matrices

2% (*m*/*V*) chitosan solution was prepared by dissolution of the appropriate amount of chitosan in 1% (*V*/*V*), acetic acid with stirring at 40 °C. After that the solution was filtered and kept overnight at room temperature to remove air bubbles. To this polymer solution, specific volumes of N-vinyl-2-pyrrolidone were added (3% and 5% *V*/*V* of the final volume) with continuous stirred for 30 min. The samples were further transferred to polystyrene petri dishes and freeze-dried. Before freeze-drying process solutions were divided in two groups and, in one of the groups, solutions were bubbled with nitrogen gas (samples of the groups will be ahead mentioned as Freeze (F) and Bubble Freeze (BF) matrices, respectively without and with N_2_ bubbling). The freeze-drying step comprised the solution freeze at −80 °C for 3 h and lyophilization for 48 h. Afterwards, small circular samples, from 5 to 10 mm in diameter, were cut, sealed under nitrogen atmosphere and gamma-irradiated. The irradiation was performed in the Precisa 22 Co-60 chamber located at the Technological Campus of Instituto Superior Técnico, Lisbon University (Bobadela, Portugal). A dose rate of 0.5 kGy·h^−1^ was used for the purpose to achieve final doses of 5 and 10 kGy. Routine Amber Perspex dosimeters (Harwell, Oxfordshire, UK) were used to monitor absorbed dose.

### 2.3. Matrices Characterization

#### 2.3.1. Scanning Electron Microscopy (SEM)

The morphology of the chitosan/PVP matrices was studied by scanning electron microscopy (SEM) in a S-2400 Hitachi microscope (Tokyo, Japan) using an accelerating voltage of 20.0 kV. Samples were previously Au/Pd coated.

#### 2.3.2. Attenuated Total Reflectance Fourier Transform Infrared Spectroscopy (ATR-FTIR)

Structural characterization was carried out by Attenuated Total Reflectance Fourier Transform Infrared (ATR-FTIR) using a micro-FTIR Thermo Scientific (Nicolet) i50 spectrometer equipped with an ATR slide-on diamond tip (Waltham, MA USA). The spectra (64 scans) were recorded at ambient temperature between 400 and 4000 cm^−1^, with a resolution of 4 cm^−1^.

#### 2.3.3. Thermogravimetric Analysis (TGA)

The thermal behavior of all specimens was assessed by thermogravimetric analysis in order to obtain information regarding the chemical/mechanical stability and robustness of the materials prepared. The thermal stability of a material is directly dependent on the nature and arrangement of their chemical bonds. The assays were carried out from 25 to 500 °C, under a nitrogen flow using a 10 °C·min^−1^ heating rate, using a TGA Q500 from TA Instruments (New Castle, PA, USA).

#### 2.3.4. Contact Angle Measurements

Contact angle measurements of the samples surface were done using a CAM 100 series 110057 goniometer (drop method). The water contact angle was measured (10 frames) during the 10 s immediately after placing the droplet. Measurements were performed in triplicate in different spots for each sample. Results are expressed as mean ± standard deviation (SD).

#### 2.3.5. In Vitro Weight Loss

The in vitro weight loss was investigated upon immersion of the matrices into physiological solution at 37 °C. Briefly, samples were dried under vacuum, weighted (*W*_0_) and immersed in the solution. After 1 and 4 days of immersion matrices were removed, washed with deionized water, dried under vacuum and weighted (*W*_deg_). The extent of degradation was calculated as the percentage of weight loss according to Equation (1):

Weight loss (%) = (*W*_0_ − *W*_deg_)/*W*_0_ × 100 (1)

All measurements were performed in triplicate and results expressed as mean ± SD.

### 2.4. In Vitro Cell Studies

As described previously [18] a Human Caucasian Fetal Foreskin Fibroblast cell line (HFFF2) was selected for the biological assays. The HFFF2 commercial cell line was obtained from the European Collection of Cell Cultures (ECACC, Salisbury, UK). These cells were used to assess the effect of chitosan-based matrices on cell adhesion and viability. For that, the cells were cultured in Dulbecco’s modified eagle medium (DMEM, Glutamax, Gibco, Waltham, MA, USA), supplemented with heat inactivated fetal bovine serum (FBS) 10% (*V*/*V*) and streptomycin and penicillin 100 U·mL^−1^ (all reagents from Gibco, Waltham, MA, USA), and incubated at 37 °C in a 5% CO_2_ humidified atmosphere. The culture medium was replace every two days. After reaching 80% confluence, the cells were trypsinized and resuspended in culture medium at a concentration of 4 × 10^4^ cell·mL^−1^ medium.

#### 2.4.1. Cell Viability Assay (almarBlue^®^)

As already mentioned before, the matrices used in this study were γ-irradiated in sealed bags. This procedure is very practical since preparation and sterilization occurred in one single step avoiding any other sterilization procedure before cellular seeding. In this way, irradiated chitosan-based matrices (Φ10 mm) were placed directly in 48-well tissue culture plate and pre-wetted with 200 μL of culture medium to improve sample’s adhesion to the bottom of the well and enable cells migration inside matrices porous structure. After 10 min 500 μL of the HFFF2 suspension (20,000 cells) were seeded in each sample and the cells cultured for 24 h at 37 °C. Control samples were established by growing cells directly on the polystyrene surface of the wells.

The cellular viability was monitored with the alamarBlue^®^ cell viability assay (Life Technologies, Bleiswijk, The Netherlands). At the desired day of culture the culture medium of each well was replaced by 300 μL of fresh culture medium supplemented with 30 μL of alamarBlue^®^ reagent and incubated for 2 h at 37 °C in a 5% CO_2_ atmosphere. The optical density (OD) of the solution collected to 96-well plate was read at 570 nm with a reference wavelength of 600 nm using a microplate reader (Tecan Spectra, Männedorf, Switzerland) and fresh culture medium was then added to the cells. The background absorbance (blank) obtained from an “empty scaffold” without cells was subtracted from the samples values. The measurements were made in triplicate and data expressed as mean ± SD. The mean optical density of the control (cells growing on polystyrene surface of wells) was set to represent 100% viability.

#### 2.4.2. Cytochemistry

After seven days of culture chitosan-based matrices and cytochemistry control samples (glass coverslips containing cells) were fixed overnight (4 °C) with paraformaldehyde, PFA 4% (in PBS), permeabilized with 0.2% Triton-X (room temperature, 10 min) and stained at room temperature for 1 h with Alexa488 conjugated Phalloidin (1:400 in PBS) Molecular Probes), to observe cell’s actin cytoskeleton. The samples were mounted on fresh PBS on a glass slide and imaged on a Leica SPE confocal system (Wetzlar, Germany). Confocal images were analyzed using Fiji, version 1.48.

### 2.5. Data Analysis and Statistics

Data are presented as the mean values ± experimental standard deviation of the mean. Values were compared between PVP content and between radiation dose using a *t*-test, with *p*-values < 0.05 considered statistically significant.

## 3. Results

### 3.1. SEM

**Figure 2:**
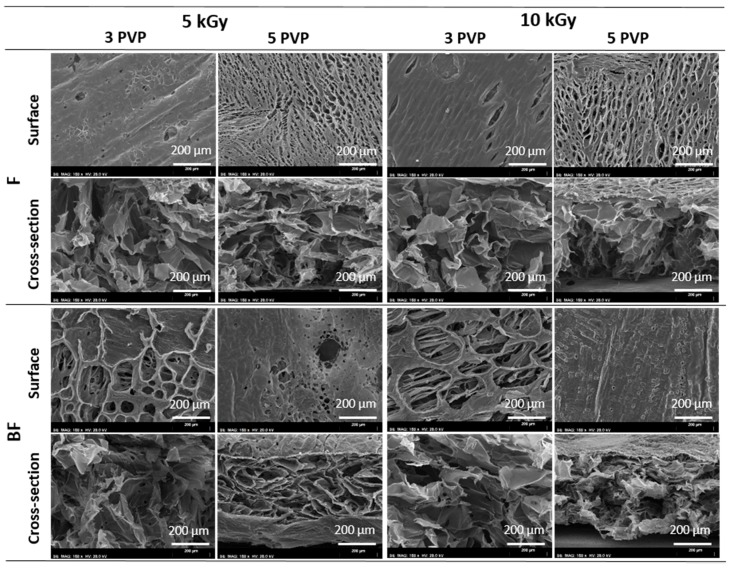
Surface and cross-sectional SEM micrographs of the chitosan/PVP matrices obtained at different experimental conditions: with and without N_2_ bubbling (BF and F), different PVP content (3% and 5%) and different radiation dose (5 and 10 kGy).

SEM representative micrographs of surface and cross-section morphology of the obtained chitosan/PVP matrices are presented in Figure 2. The images point out that PVP content, radiation dose and the bubbling of N_2_ are important parameters that lead to significant differences at matrices surface.

The increment of PVP content and of the absorbed radiation dose apparently leads to different high dense materials with more homogeneous surfaces with the consequent decrease of their roughness. By other way, bubbling of N_2_, particularly for the lower PVP content in study (3%) gives rise to materials with a rougher surface with more porous and open structures. Additionally, despite the different surface morphology, all matrices exhibit an inner interconnected porous structure.

### 3.2. ATR-FTIR

**Figure 3:**
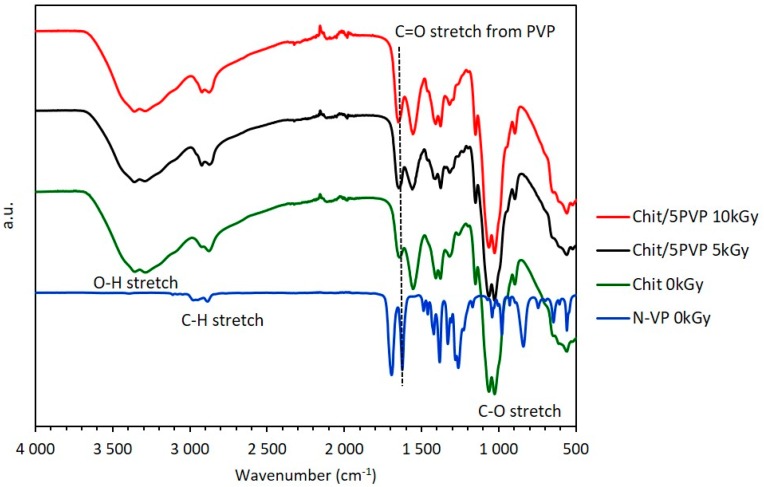
ATR-FTIR spectra of pristine N-vinyl 2-pyrrolidone (N-VP), pristine chitosan and chitosan/PVP (5% PVP; N_2_ bubbling) matrices irradiated at different doses.

ATR-FTIR spectra of chitosan and chitosan/PVP matrices at different radiation doses were obtained and representative results are shown in Figure 3. The observed broad peak at around 3350 cm^−1^ is assigned to the stretching vibrations of O–H and N–H groups from chitosan. Other characteristic peaks of chitosan also appears at about 1636 cm^−1^ (amide I band), 1559 cm^−1^ (amide II, N–H deformation mode) and 1328 cm^−1^ (amide III). Absorption peaks at 1142 cm^−1^ (antisymmetric elongation of the C–O–C bridge) and 1085 cm^−1^ (C–O ring elongation) are also characteristic of the chitosan saccharide structure [21]. The peak in the 2800–2900 cm^−1^ region shows stretching vibration of C–H bonds common of both chitosan and PVP. On the other hand, the increase in the intensity peak at C=O stretch region, 1624 cm^−1^, confirms the PVP presence in the irradiated matrices [22]. The similarity of peak intensity at 895 and 1151 cm^−1^ also characteristic of the saccharide structure, suggest that the chitosan β-glucosidic linkages remains stable after γ-irradiation within the studied conditions.

### 3.3. TGA

**Figure 4:**
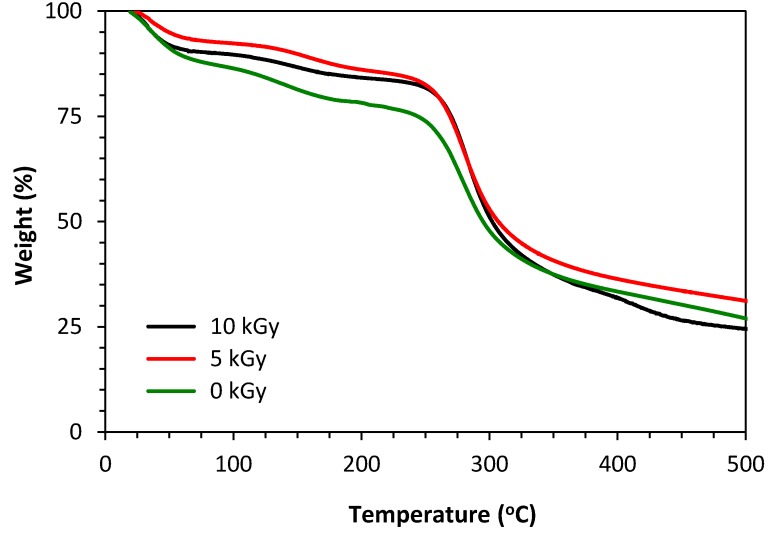
TGA thermograms of non-irradiated and γ-irradiated chitosan/PVP matrices (3% PVP; BF) achieved.

The TGA thermograms of non-irradiated and γ-irradiated chitosan/PVP matrices are presented in Figure 4. The depicted thermal profiles are representative of both preparation methodologies in study independently of PVP content. Data show an initial weight loss in all matrices up to 100 °C, which is usually associated with the loss of water sorbed by, or trapped in, the samples. They also show a second, more pronounced mass loss starting at about 250 °C for all irradiated and non-irradiated matrices. This variation is associated with the degradation of the saccharide structure of the molecule, including the decomposition of chitosan deacetylated (and acetylated) units. However, it is also observable that from 400 °C the matrices irradiated at 10 kGy suffer a faster weight loss than those not irradiated and irradiated at 5 kGy. This can be attributed to the PVP depolymerization due to thermal degradation [23].

### 3.4. Contact Angle Measurements

**Figure 5:**
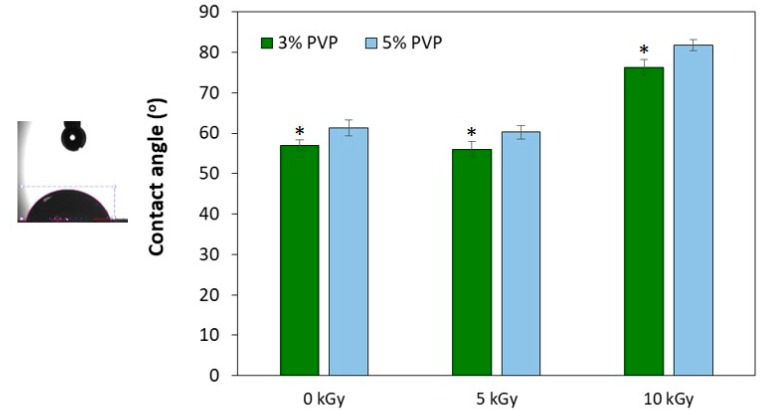
Contact angle of BF chitosan/PVP matrices (N_2_ bubbling). Data expressed as mean ± SD (*n* = 3). Contact angle does not differ significantly between radiation doses of 0 and 5 kGy (* means statistically significant with *p* < 0.05).

The surface hydrophilicity was assessed by measuring the contact angle through water spread of a droplet on the matrices’ surface. Figure 5 evidences the hydrophilic character of chitosan/PVP matrices obtained by γ-irradiation since all exhibit values lower than 90° and suggesting a great water absorption capacity. Nevertheless it can be observed that a radiation dose of 10 kGy lead to significant higher contact angle values when compared to a dose of 0 or 5 kGy, meaning a less hydrophilic surface, probably due to the decrease of intermolecular interactions of a more crosslinked structure. These results are in accordance with ATR-FTIR and TGA data that suggest an improved structural stability at 10 kGy due to radiation-crosslinking. A higher PVP content also leads to slightly significant higher contact angle values.

**Figure 6:**
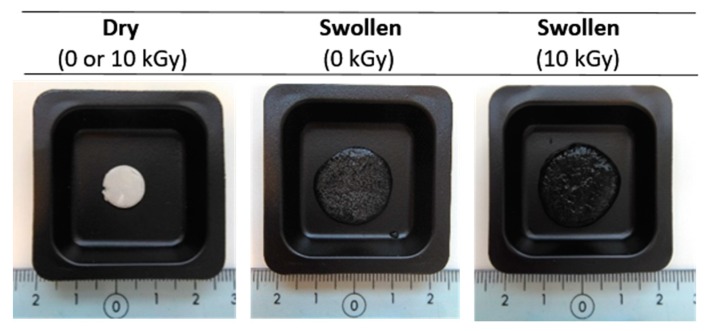
Macroscopic view of non-irradiated and γ-irradiated BF chitosan/PVP matrices in dry and swollen state.

Although quantitative swelling studies were not performed, macroscopically it was possible to observe high swelling capacity of samples in water at 37 °C (Figure 6). Since macroscopically appearance of non-irradiated and γ-irradiated dry samples are the same, just one image is shown.

### 3.5. In Vitro Weight Loss

**Figure 7:**
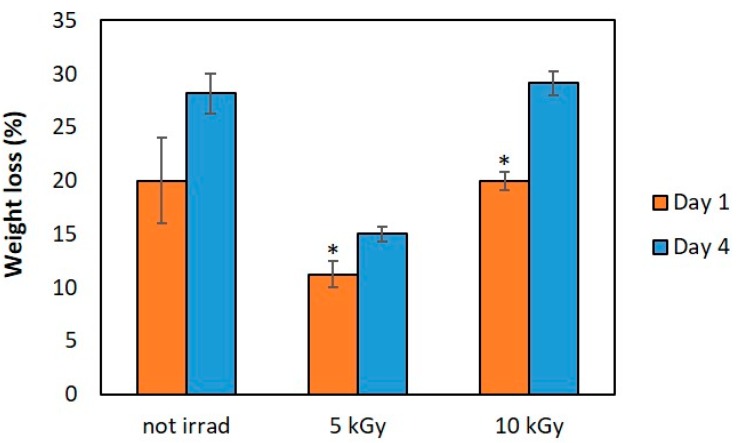
In vitro weight loss of BF chitosan/PVP matrices with 5% PVP content in physiological solution. Data expressed as mean ± SD (*n* = 3; * *p* < 0.05).

Figure 7 shows the effect of γ-radiation dose on matrices weight loss in physiological medium. When compared with the values of non-irradiated matrices, which do not differ significantly within the period studied, it is noted a decrease in the weight loss of matrices irradiated at 5 kGy particularly significant after 4 days. This suggests an increase in matrices stability due to radiation crosslinking. However, for a radiation dose of 10 kGy it seems that radiation scission effect overlaps the crosslinking effect, leading to higher degradation values than the ones observed at 5 kGy although identical to the ones of non-irradiated matrices.

### 3.6. Cellular Viability

The cytotoxicity of the chitosan/PVP matrices was assessed using in vitro cell-based assays with Human Caucasian Fetal Foreskin Fibroblast (HFFF2) as cell model and alamarBlue^®^ to determine the cell viability. HFFF2 is a line of human dermal fibroblasts primary cells, therefore they are cells of human origin that were collected form the tissue under study. Moreover, due to their low-passage number, the HFFF2 used in this study should recapitulate the function of the in vivo native dermal tissue.

**Figure 8:**
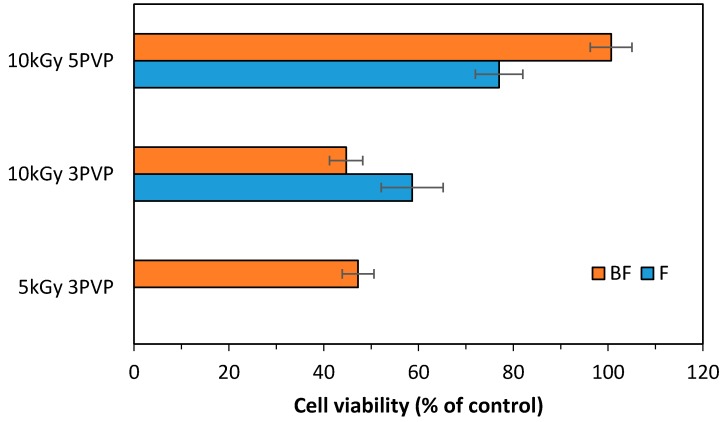
In vitro cellular viability (mean ± SD, *n* = 3) at day 1 of HFFF2 cultured on chitosan/PVP (radiation dose, 5 and 10 kGy; PVP content, 3% and 5%; methodology, with and without N_2_ bubbling, BF and F respectively).

HFFF2 cells adhered and remain viable in all irradiated matrices, at least during one day of culture (see Figure 8). However BF chitosan/PVP matrices with 5% PVP and irradiated at 10 kGy, lead to higher fibroblast adhesion and viability rates.

**Figure 9:**
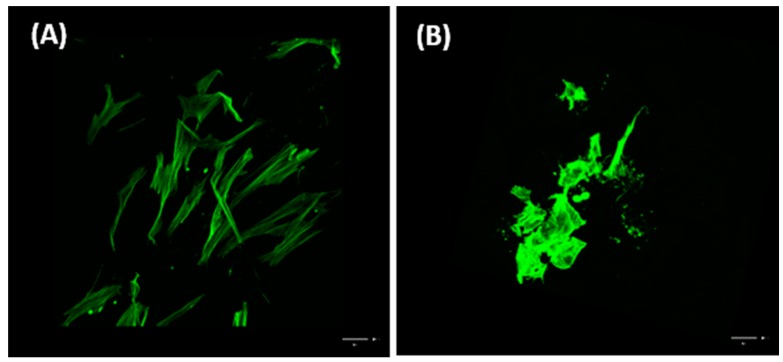
HFFF2 cells growing for 7 days in culture on 10 kGy, N_2_ bubbled, 5% PVP membranes (**B**) display a different morphology and a very dense actin cytoskeletal organization, as compared to control cells growing on the polystyrene surface of the wells (**A**) but are able to invade the depth of the membrane (green: actin staining; scale bar 50 µm).

Analyzing the viability rates, at least on day 1 of culture, we verified that the viability of the fibroblasts was higher on chitosan/PVP prepared with a radiation dose of 10 kGy and 5% PVP with N_2_ bubbling. After a few days of culture, it was difficult to maintain the membranes adherent to the bottom of the wells and the measurements of cell viability were no longer accurate since cells could be growing on the bottom plastic surface of the wells. Nevertheless, we analyzed cells growing for 7 days on these membranes and imaged the morphology of these fibroblasts (see Figure 9). We could observe that in the membranes, cells displayed a denser morphology than that of control cells growing on polystyrene, as assessed by the actin microfilaments staining intensity and the cell shape. Cells were imaged across a *z* range and the figure is a maximum intensity projection of the obtained *z* stack, therefore we could conclude that cells are able to invade the matrix in 3D (see Figure 9)

## 4. Discussion

The general approach of this study has been to prepare by alternative technologies like γ-radiation, porous biocompatible and biodegradable chitosan based matrices to be used as skin scaffolds.

SEM images point out that despite all matrices exhibit an inner interconnected porous structure, its surface morphology is dependent on PVP content, radiation dose and the bubbling of N_2_ during homogenization step. Moreover, the preliminary cell-scaffold interaction study shows that within the preparation conditions tested, cells seem to prefer more homogeneous surfaces with lower pores dimensions, which can be achieved by an appropriate selection of the above mention parameters. Thus, since the surface properties, both chemical and topographical characteristics, play an important role in terms of cell adhesion, scaffold-cell interaction and tissue organization [1,24], PVP content, radiation dose and the bubbling of N_2_ during homogenization step can be used to tailor the matrices’ surface and promote a better adjust of the matrices (topography and chemically) to its use as skin scaffolds.

ATR-FTIR analysis was used to detect the presence of PVP groups as well as to determine the effect of irradiation dose on matrices molecular structure. The presence of the previously mentioned characteristic peaks for chitosan and PVP confirms the occurrence of chemical crosslinking between chitosan and NVP molecules when γ-radiation exposed (within the studied dose range and dose rate). These results are in accordance not only with TGA data but also with authors previous study with chitosan and poly(vinyl alcohol) [19], where the presence of a crosslinking polymer type seems to act as a radiation shield leading to an improvement in structural stability, despite the degradative type behavior of chitosan when γ-irradiated. Moreover, the absence of chemical initiators and the possibility of have in one step functionalization and sterilization [19], makes this radiation procedure a very promising possibility to obtain chitosan based matrices to be used as reliable and efficient skin scaffolds.

The wettability of the matrices is an important feature that can influence cell adhesion and colonization inside the structure. As the contact angles of all samples tested were less than 90°, it can be stated that all materials have a hydrophilic character. Both radiation doses studied, led to the obtaining of structurally stable hydrophilic matrices, with 10 kGy irradiated matrices having a higher mass loss by hydrolytic degradation after 4 days (about 30%). However, despite matrices might be seen as too fragile, it is expected that its degradation rate follows the skin regeneration rate. Even that in vitro weight loss behavior be influenced by at least two radiation effects, crosslinking and scission, results show a tendency which will be evaluated and optimized regarding matrices long-term durability versus cell viability in order to be used as skin scaffold. This will be the next step of the study.

Results from in vitro cell studies evidence that all matrices displayed biocompatibility, i.e., they are not toxic to the cells allowing the attachment, growing and maintenance of fibroblasts in all of obtained matrices. Nevertheless, growth is limited and cell densities are low and difficult to assess over the days of culture. However, some improvements have been achieved as compared to previous works using chitosan/poly(vinyl alcohol) [19]. The most promising procedure, out of the ones that were tested, seems to be the bubbling of the polymer solution during the homogenization step, followed by freeze-drying and a γ-irradiation dose up to 10 kGy. The bubbling procedure might confer favorable porosity characteristics (e.g., interconnection, dimension) to the membrane that allows the cells to penetrate and colonize in a more efficient way the scaffold. Although these preliminary data are promising, further modifications to introduce multiple cell-binding ligands in the matrices could be considered in order to improve cell affinity for the matrices and to achieve a better morphology and behavior, namely cell proliferation, of the fibroblasts growing on these scaffolds.

## 5. Conclusions

The current study allowed to clarify the correlation between the different preparation conditions and the final properties of the chitosan/PVP matrices. Different matrices composition led to structurally stable hydrophilic and biocompatible matrices, with the matrix chitosan/PVP (5%), BF and γ-irradiated at 10 kGy, presenting the higher cellular viability. The results evidence that matrices physicochemical features and consequent biological behavior are dependent on methodology used, composition and radiation dose. Once can thus say that significant progress was reached in the development by γ-irradiation of biocompatible and biodegradable chitosan/PVP matrices to be used as reliable and efficient skin scaffolds. The main outcome concerns the identification of the best configurations to be used at animal testing, and the preparation protocol definition of the crosslinked structure for optimized stability and behavior to mimic the required structural and biological functions.
